# Long-term scarring outcomes and safety of patients treated with NovoSorb^Ⓡ^ Biodegradable Temporizing Matrix (BTM): An observational cohort study

**DOI:** 10.1016/j.jpra.2023.05.003

**Published:** 2023-05-28

**Authors:** C.H. Lo, M.J.D. Wagstaff, T.M. Barker, L. Damkat-Thomas, S. Salerno, D. Holden, E. Concannon, K. Heath, P. Coghlan, H. Cleland

**Affiliations:** a Victorian Adult Burns Service, The Alfred, 55 Commercial Rd, Melbourne, VIC 3004, Australia; b Department of Surgery, Central Clinical School, Monash University, 99 Commercial Rd, Melbourne, VIC 3004, Australia; c Adult Burns Service and Department of Plastic and Reconstructive Surgery, Royal Adelaide Hospital, Port Rd, Adelaide, SA 5000, Australia; d PolyNovo Biomaterials Pty Ltd, 2/320 Lorimer Street, Port Melbourne, VIC 3207, Australia

**Keywords:** Biodegradable Temporizing Matrix, Biodegradable polyurethane, Dermal regeneration template, Burn

## Abstract

**Background/Aim:**

NovoSorb^Ⓡ^ Biodegradable Temporizing Matrix (BTM) is a relatively novel, biodegradable polyurethane-based dermal regeneration template. The aim of this study was to evaluate the long-term scarring outcomes and safety of BTM in patients who underwent dermal reconstruction involving ≥5% of the total body surface area.

**Methods:**

This was a postmarket, multicenter, observational cohort study involving evaluation of long-term outcomes in patients treated with BTM. A total of 55 patients (35 from Royal Adelaide Hospital, South Australia, and 20 from Victoria Adult Burns Service, The Alfred, Victoria) who underwent dermal repair with BTM between 2011 and 2017 were screened for inclusion in this study. All patients had BTM implanted for ≥18 months.

**Results:**

Fifteen eligible patients with a mean (SD) age of 49.1 (14.3) years completed study assessments. These patients had a total of 39 areas treated with BTM. Using the Patient and Observer Scar Assessment Scale, scar quality was reported to be good by both observers and patients, with a mean (SD) observer score across all lesions of 3.6 (1.2) and mean (SD) overall opinion of 3.8 (1.2) as well as a mean (SD) patient score of 3.5 (1.2) and overall opinion of 5.0 (2.2). No adverse events or adverse device effects were reported or identified.

**Conclusion:**

The long-term scar quality is comparable to published studies. BTM is safe in the long term with no additional risks or adverse consequences being identified.

## Introduction

Dermal regeneration templates restore or reconstruct the dermal layer of the skin. Derived from biological, synthetic, or a combination of materials, a number of these products are commercially available in the market. However, no 1 template possesses all desired properties in a single 3-dimensional construct.^1^, ^2^, ^3^ Some of these properties include a porous structure suitable for cell and tissue penetration, controllable biodegradation rates, low toxicity and immunogenicity, and good structural integrity for structural maintenance during degradation.^4^

NovoSorb^Ⓡ^ Biodegradable Temporizing Matrix (BTM) (PolyNovo Biomaterials Pty Ltd, Port Melbourne) is a relatively novel, biodegradable polyurethane-based dermal regeneration template.^5^ Fully synthetic, BTM is available in numerous countries for various wound types, including Australia, where it is indicated for use in the treatment of full or deep partial-thickness burns, surgical and reconstructive wounds, and traumatic wounds. Histologic assessments performed at 12 months detected microscopic remnants of the polymer, surrounded by giant cells.^6^^,^^7^ Hydrolysis and degradation of the polymer were largely complete, and nonhydrolyzable fragments were phagocytosed.

The aim of this prospective clinical study was to evaluate the long-term scarring outcomes and safety of BTM in patients who underwent dermal reconstruction involving ≥5% of the total body surface area (TBSA). The effectiveness of BTM as a wound temporizer and its ability to achieve wound closure has already been proven.^5^, ^6^, ^7^ However, to our knowledge, this is the first study to focus on long-term scar quality outcomes of BTM. Evaluating patient outcomes beyond the anticipated 12 to 18 months of lifetime of BTM would also determine if new, previously unidentified risks are introduced that may require control or mitigation at a later stage.

## Methods

### NovoSorb^Ⓡ^ BTM

BTM has been previously described.^5^ Briefly, it is composed of 2-mm thick, biodegradable polyurethane matrix bonded via a polyurethane adhesive layer to a superficial transparent nonbiodegradable polyurethane sealing membrane 150 µm in thickness.^8^^,^^9^ The white, open-cell matrix has a high degree (90%) of porosity (cell size, 100-500 µm), providing a scaffold for dermal tissue integration, and biodegrades *in vivo* after 12 to 18 months.^6^^,^^7^^,^^10^ The temporary bonding or adhesive polyurethane layer retains the sealing membrane on the dermal component until delamination is performed. This provides flexibility for staging subsequent procedures, and the sealing membrane has been reported as remaining in place for up to 77 days *in vivo*.^11^ At a second operation, the sealing membrane is removed and the wound definitively closed using the clinician's preferred method, including a split skin graft.

### Study design

This was a postmarket, multicenter, observational cohort study involving evaluation of long-term outcomes in patients treated with BTM. The study received ethics approval from the Gold Coast Hospital & Health Service Human Research Ethics Committee (study reference: HREC/2019/QGC/57141), providing approval at the study sites under the Australian National Mutual Acceptance of ethics review for multicenter clinical trials. The study was registered prospectively on the Australian & New Zealand Clinical Trials Register (www.anzctr.org.au; reference number, ACTRN12619001162101) and performed in accordance with the standard EN ISO 14155 Clinical investigations with medical devices on human participants – Good clinical practice and Recommendations guiding physicians in biomedical research involving human participants.

### Study population

A total of 55 patients (35 from Royal Adelaide Hospital, South Australia, and 20 from Victoria Adult Burns Service, The Alfred, Victoria) who underwent dermal repair with BTM between 2011 and 2017 were screened for inclusion in this study. The patients may have received treatment with BTM on several areas and for treatment of various types of wounds, including burn injuries and surgical wounds. Hospital records at each study site were reviewed to identify potential study subjects. All patients ultimately enrolled as study subjects met all the following inclusion criteria: (1) had BTM implanted for a period of ≥18 months, (2) area treated with BTM involved ≥5% of TBSA, and (3) gave written informed consent to participate in the study.

### Study endpoints

The data collected included patient demographics, wound etiology, details of anatomic areas and area treated with BTM, medical history, and current health status. All study subjects attended a single clinical visit to perform a physical examination and scar assessment. Scarring outcomes for each area treated with BTM were assessed using the Patient and Observer Scar Assessment Scale (POSAS).^12^^,^^13^ The POSAS includes 2 separate but complementary components that assess scar quality from different perspectives. Each item has a 10-point score: the patient scale contains 6 items (range, 6-60) and the observer scale contains 5 items (range, 5-50). On the patient scale (pain, itch, color, stiffness, thickness, and regularity), the lowest score of 1 corresponds to normal skin, whereas a score of 10 reflects the worst possible scar or sensation. All observer scale assessments (vascularization, pigmentation, thickness, relief, and pliability) were performed by a surgical physician or other delegated clinical research staff members.

The safety of BTM implantation was evaluated by the incidence of adverse events (AEs) or adverse device effects (ADEs) occurring ≥18 months after BTM application. An AE was defined as any untoward medical occurrence, unintended disease or injury, or untoward clinical signs (including abnormal laboratory findings) in patients. An ADE was defined as an AE related to the use of BTM, including AEs resulting from insufficient instructions for use or any malfunction of BTM or any event resulting from use error or intentional misuse of BTM.

### Data handling and analysis

No sample size was calculated for this postmarket clinical follow-up. Data were collected from all patients who fulfilled inclusion criteria and attended the study visit. Descriptive analyses were completed for this study. The continuous parameters included number of observations, mean, median, SD, minimum, and maximum. Categorical parameters were summarized as frequency counts and percentages. Scar quality as measured using POSAS were reported at the time of assessment. The POSAS scores were reported as mean, median, SD, 95% confidence interval (CI), minimum, and maximum. All CIs reported were 2-sided 95% CIs, unless otherwise stated.

## Results

**Figure 1:**
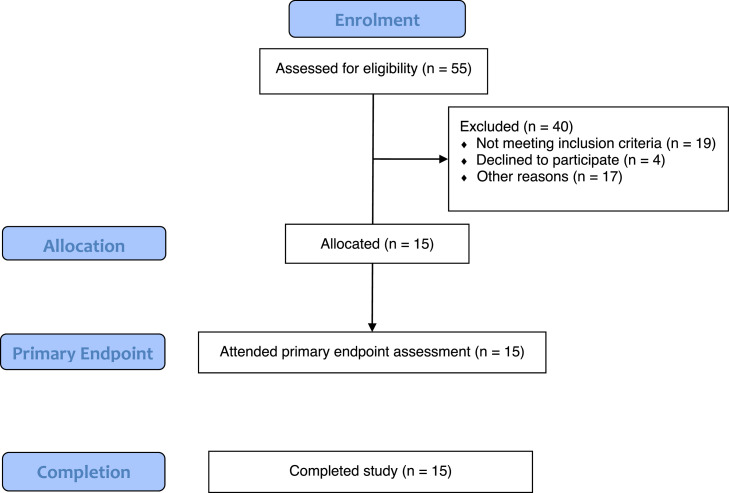
Flow chart of enrollment process with 15 patients completing the study. *other reasons for exclusion from study (n=17) included patient being uncontactable, previous patient request for no further contact, failure to attend assessments, and patient death.

Fifteen eligible patients with a mean (SD) age of 49.1 (14.3) years attended assessment visits and completed study assessments (Figure 1, Table 1). Thirteen of these patients (86.7%) sustained burn injuries. A total of 39 areas were treated with BTM; 25 had subcutaneous fat only (64.1%) as the wound bed, 11 areas had a fascial wound bed (28.2%), and 3 areas (7.7%) had a mixed wound bed (2 with exposed bone) (Table 2). Eleven areas treated with BTM involved the upper limbs only (28.2%), 17 areas involved the lower limbs only (43.6%), 4 areas involved the trunk only (10.3%), 1 area involved the head and neck region only (2.6%), and 6 areas involved multiple regions (15.4%). Ten patients (66.7%) had at least 2 areas treated with BTM, and 2 patients (13.3%) had 5 areas treated. Assessment of 1 area was inadvertently omitted or not recorded, leaving a total of 38 areas treated using BTM that were evaluated (Tables 3 & 4).

**Table 1:** Summary of demographics of patients included in the study.

Parameter	All patients (n = 15)
Age (years)	
Mean (SD)	49.1 (14.8)
Median	50.0
Minimum-maximum	26-80
Sex	
Female	2 (13.3%)
Male	13 (86.7%)
Race	
Asian	2 (13.3%)
Caucasian	13 (86.7%)
Wound etiology, n (%)	
Full-thickness burns	13 (86.7)
Necrotizing fasciitis	2 (13.3)
Secondary burn scar reconstruction*	1 (6.7)

⁎ 1 patient had 3 areas treated with Biodegradable Temporizing Matrix (1 burn scar reconstruction, 2 full-thickness burns).

**Table 2:** Summary of underlying pathology and BTM application.

Extent of areas treated with BTM (mean [range]) (% TBSA)	
All injuries treated with BTM	25.2 (5-65)
Burn injuries treated with BTM	27.9 (9-65)
Necrotizing fascitis patient 1	5
Necrotizing fascitis patient 2	5.5
Secondary burn scar contracture reconstruction	5
Areas treated with BTM	
Total	39
Full-thickness burns	36
Necrotizing fascitis	2
Secondary burn scar reconstruction	1
Time from BTM application to assessment date (months)	
Mean (SD)	56.0 (15.2)
Median	53.2
Range	18.5-84.9

**Table 3:** POSAS scores of individual items (allowable range, 1-10) in 38 BTM-treated areas evaluated in 15 patients (1 = normal skin, 10 = worst scar imaginable).

	Mean (SD)	95% CI	Median	Minimum	Maximum
Observer scale					
Vascularization	3.4 (1.2)	3.0-3.8	3	1	6
Pigmentation	3.3 (1.5)	2.8-3.8	3	1	7
Thickness	3.0 (1.3)	2.5-3.4	3	1	7
Relief	3.4 (1.8)	2.8-4.0	3	1	9
Pliability	3.4 (2.0)	2.8-4.1	3	1	9
Surface area	2.9 (1.8)	2.3-3.5	3	1	7
Patient scale					
Pain	1.4 (0.6)	1.2-1.6	1	1	3
Itch	1.9 (1.0)	1.6-2.3	2	1	5
Color	4.2 (4.2)	3.5-4.9	4	1	10
Stiffness	4.3 (2.6)	3.5-5.2	3	1	10
Thickness	3.8 (2.3)	3.0-4.5	3	1	10
Relief	4.1 (2.2)	3.4-4.9	4	1	10

**Table 4:** POSAS cohort mean score and overall opinion in 15 patients included for analysis (1 = normal skin, 10 = worst scar imaginable).

	Mean (SD)	95% CI	Median	Minimum	Maximum
Observer scale					
Mean score*	3.6 (1.2)	3.0-4.2	3.1	1.7	6.3
Overall opinion⁎⁎	3.8 (1.2)	3.2-4.4	3.5	2.0	6.0
Patient scale					
Mean score	3.5 (1.2)	2.8-4.1	3.4	1.9	5.8
Overall opinion	5.0 (2.2)	3.8-6.2	5.0	2.2	8.0

⁎ Cohort mean score calculated by averaging mean score per subject (n = 15).

⁎⁎ Cohort mean overall opinion calculated by averaging the mean overall opinion per subject.

**Figure 2:**
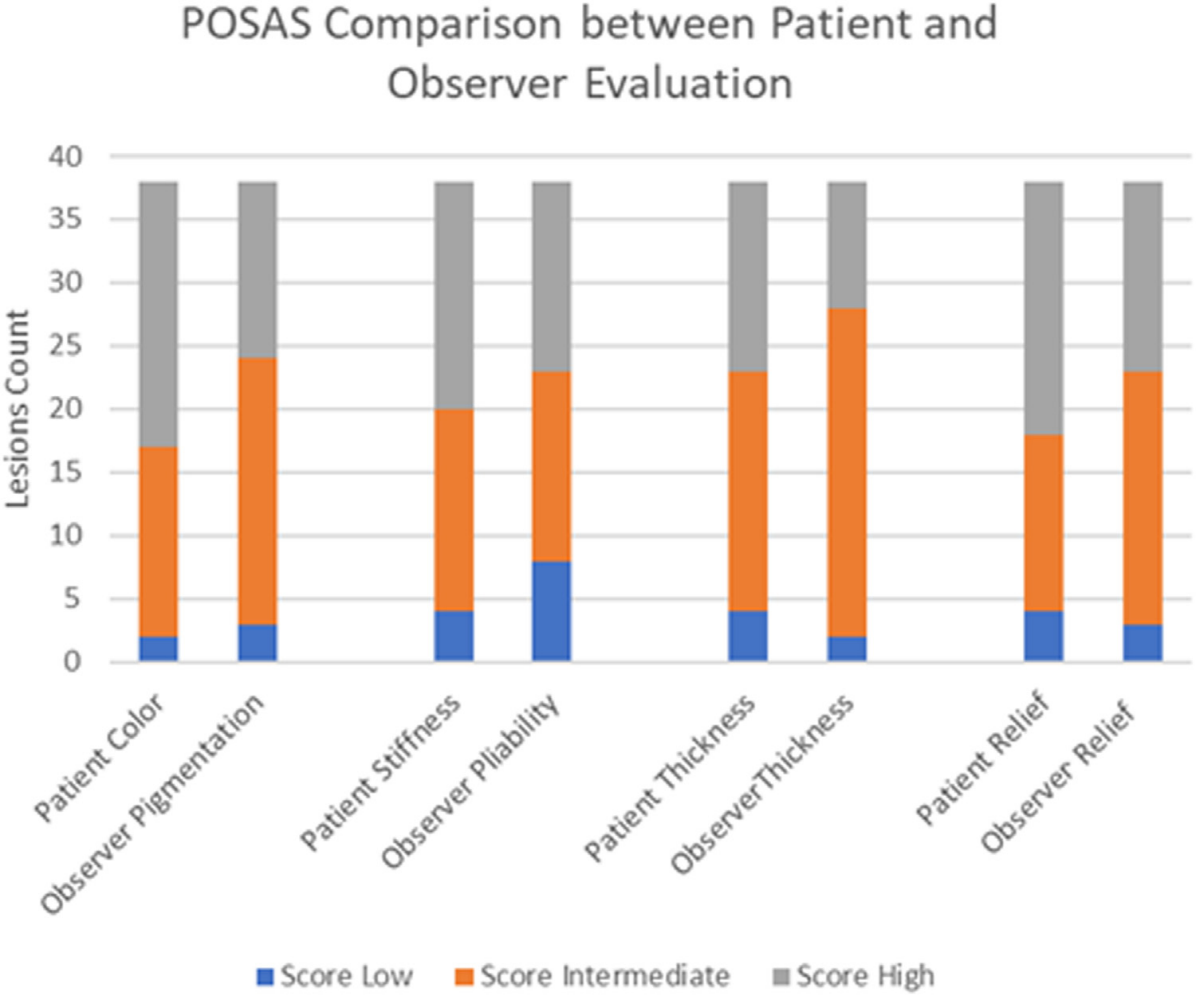
Comparison of matched patient and observer scores arbitrarily grouped into 3 categories described as low, intermediate, and high scores.^14^ For all 4 parameters, patient scores were higher than observer scores (closer to normal skin). * scores defined as (1) low score: no difference with normal skin, item score =1 (2) intermediate score: minor differences with normal skin, item score = 2 or 3 (3) high score: major differences with normal skin, item score ≥ 4.

**Figure 3:**
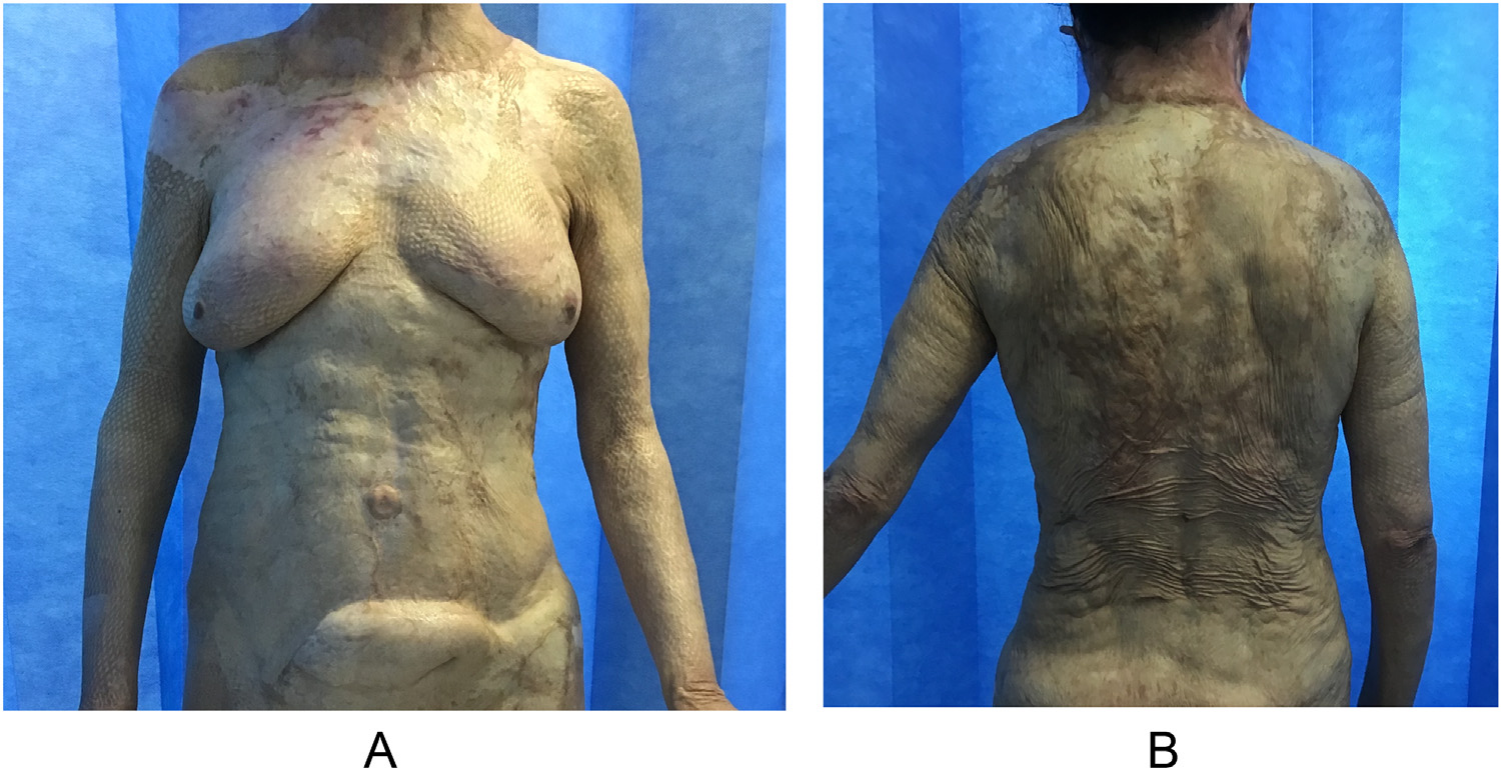
A 33-year-old woman who experienced 70%-TBSA flame burns and inhalational injury underwent staged debridement, BTM application and autologous grafting. Photographs taken of (a) the anterior aspect of the trunk and (b) posterior aspect of the trunk approximately 4 years and 2 months after surgery demonstrated fair results, with a POSAS observer mean score of 4.2 and patient mean score of 4.8.

**Figure 4:**
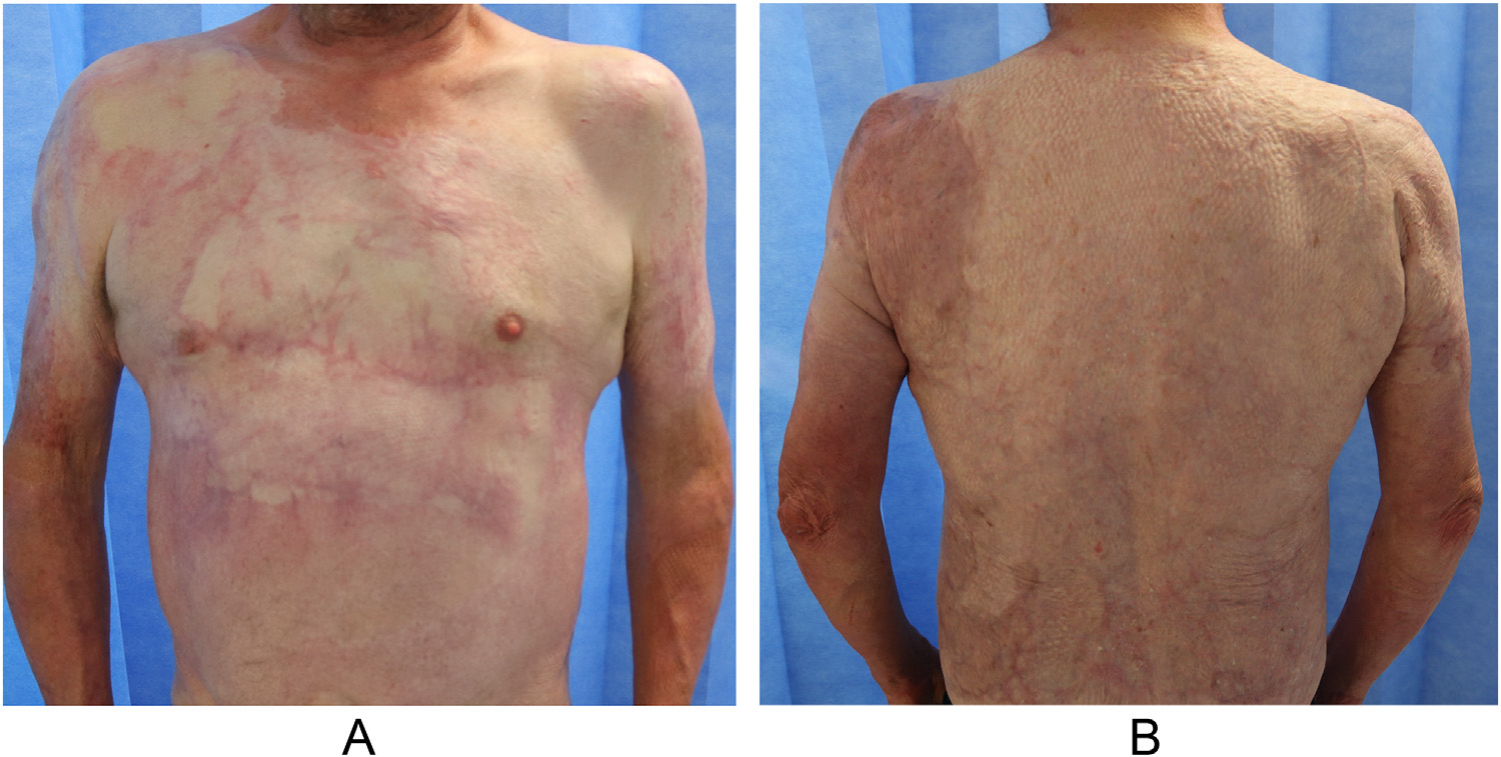
A 55-year-old man experienced 73%-TBSA flame burns involving petrol accelerant. At approximately 7 years after injury, photographs taken of (a) anterior aspect of the trunk and (b) posterior aspect of the trunk demonstrated good results, with a POSAS observe mean score of 1.7 and patient mean score of 2.0.

**Figure 5:**
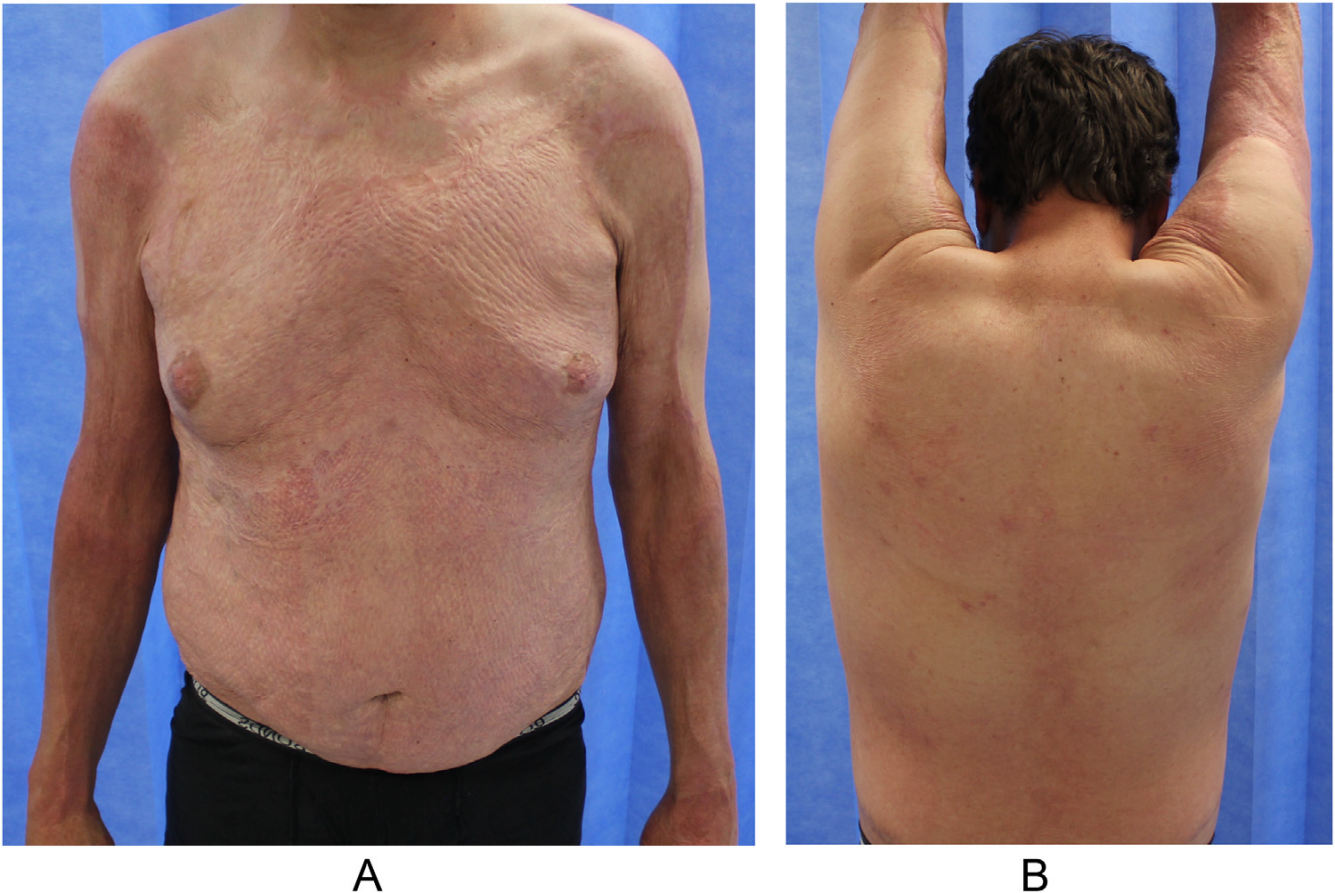
A 31-year-old man who experienced 71%-TBSA chemical and flame burns underwent staged BTM application and grafting of the right side of the anterior aspect of the trunk, right side of the back, and right upper limb. Photographs taken at 4 years and 6 months of (a) the anterior aspect of the trunk and upper limbs and (b) posterior aspect of the trunk with arms elevated demonstrated good results, with a POSAS observe mean score of 2.7 and patient mean score of 2.7.

Based on the individual parameters of POSAS, a greater proportion of the lesions examined in the study showed low-to-intermediate scores of vascularity, pigmentation, thickness, relief, pliability, and surface area evaluated by the observer and intermediate scores for color, stiffness, thickness, and relief evaluated by the patient (Table 3). The patients’ parameters of pain and itch had both low-to-intermediate scores, indicating a perception of the scar being closer to normal skin. Long-term (>18 months) scar quality was reported as good by both observers and patients, with a mean (SD) observer score across all lesions of 3.6 (1.2) and mean (SD) overall opinion of 3.8 (1.2) as well as mean (SD) patient score of 3.5 (1.2) and overall opinion of 5.0 (2.2) (Table 4, Figure 2, Figure 3, Figure 4, Figure 5).

No AEs or ADEs were reported or identified.

## Discussion

Publications on appraisal of long-term scarring outcomes are limited, but the results of this study were comparable to the few reported in the literature.^14^ A multicenter Dutch study assessed 251 patients with severe burn injuries with a follow-up of >5 years after injury.^14^ In the Dutch study, the mean (SD) POSAS patient score was 3.4 (2.0) compared with 3.5 (1.2) in the current study, and the mean (SD) patient overall opinion score was 4.1 (2.6) compared with 5.0 (2.2) in the current study.^14^ Another study that examined patients with burn scars of maximum 20% TBSA ≥18 months after injury found lower scores than those reported in the current study.^15^ Keeping in mind that the lowest score of 1 corresponds to normal skin, the mean (SD) POSAS observer score was 1.94 (0.78) compared with 3.6 (1.2) in this study, with a mean observer overall opinion score of 2.34 (1.04) compared with 3.8 (1.2) in this study. However, the mean percentage of TBSA of the burn lesions evaluated was significantly lower (5.2%) than that reported in the current study (25.2%).^15^ It is expected that patients with more severe burns (higher percentage of TBSA) will have higher POSAS scores, representing worse scar quality.^16^

POSAS is a reliable and complete scar evaluation tool that incorporates patient-reported outcome measures and patients’ perspective on their scars.^12^^,^^15^ In this study, 86.7% of the population cohort sustained burn injuries. Only scar assessment scales with a subjective component are able to capture patient perceptions of scarring as well as pain and itch.^12^^,^^17^ Even authors of the Vancouver Scar Scale, the most frequently applied tool for burn scars, admitted to deficiencies of Vancouver Scar Scale in capturing important data, including pain and itch.^13^ Although the observer scale is expected to be more accurate (because of training and experience), the patient scale remains a relevant criterion because it reflects patients’ perspectives on their scar quality. An individual patient's perspective can differ from the clinician perspective and is a very important component of such studies because patients have to live with their scars, and their perspectives drive future scar management measures.^14^ Although the patient scale and observer scale are 2 separate instruments measuring scar quality from different perspectives, several of the parameters are aligned and are amenable to comparison (Figure 2). For these qualities, patients tend to give higher scores (poorer scar quality), suggesting that patients are more aware or critical of the final appearance of their scar and what may be considered socially acceptable (Figures 3 & 4). Other factors, such as female sex, have also been associated with poorer overall opinion of scarring.^14^

The findings of this study are consistent with those reported by other early authors on the use of BTM in the management and treatment of patients with severe burn injuries. The first prospective pilot study to investigate the efficacy of BTM in 5 patients with large, full-thickness burns (20%-50% TBSA) included gaining experience with the first-in-human application of BTM for severe burn indications and assess clinical outcomes at 12 months following injury.^18^ In this study, assessment of scar quality was limited to a single assessment at 12 months after injury for 3 out of 5 patients using the POSAS. The individual POSAS observer overall opinion score was reported as 2, 4, and 2 and the patient overall opinion as 2, 6, and 3 for the 3 patients.^18^

No AEs and no ADEs were recorded or identified in the study. All long-term treatments (>18 months after BTM application) on the BTM-treated sites were confirmed as not being BTM related or were part of anticipated treatment course in patients who had extensive full-thickness injuries. All treatments and interventions after BTM application were evaluated as being expected and consistent with the clinical standard of care for such patients, eg, laser scar treatment, contracture release, other scar management procedures.

This study has some limitations. The number of study participants was relatively low, with 15 subjects evaluated at participating sites. The COVID-19 pandemic and associated restrictions affected and delayed the conduct of this study, limiting the time available for patient enrollment, assessment, and data collection. The study population was predominantly male Caucasian and presented with full-thickness lesions due to severe burn injuries and necrotizing fascitis. A range of factors are known to affect scar outcomes after injury, including sex, age, Fitzpatrick skin type, race, extent of injury, time to heal, and need for grafting.^16^ Single POSAS observer scoring was performed for each lesion, although the use of multiple observers has been shown previously to improve interobserver reliability.^12^ In this study, neither information on wounds treated with other dermal matrixes was collected nor direct comparisons were made with other wound closure methods. Long-term outcomes with regard to wound contracture are the subject of an ongoing study.

## Conclusion

The results of this study indicate that BTM is safe in the long term, with no additional risks or adverse consequences being identified. In addition, the long-term scar quality assessed using POSAS is comparable with that in published studies. This study adds further evidence to the effectiveness and safety of BTM as a dermal regeneration template for a range of conditions, including severe burns and necrotizing fascitis. BTM is a useful addition to the armamentarium of burn surgeons as well as plastic and reconstructive surgeons.

## Ethics Approval Statement

The study received ethics approval from the Gold Coast Hospital & Health Service Human Research Ethics Committee (study reference: HREC/2019/QGC/57141), providing approval at the study sites under the Australian National Mutual Acceptance of ethics review for multicenter clinical trials.

## Declaration of Competing Interest

The authors declare the following financial interests or relationships which may be considered as potential competing interests. Heather Cleland and Cheng Hean Lo were investigators in another study (The Alfred HREC Number: 202/15) sponsored by PolyNovo Biomaterials Pty Ltd. Marcus Wagstaff serves as a consultant for and holds shares in PolyNovo Biomaterials Pty Ltd. Timothy Barker is an employee of PolyNovo Biomaterials Pty Ltd. All remaining authors have declared no conflicts of interest.
